# Latest Insights into Marek’s Disease Virus Pathogenesis and Tumorigenesis

**DOI:** 10.3390/cancers12030647

**Published:** 2020-03-10

**Authors:** Luca D. Bertzbach, Andelé M. Conradie, Yu You, Benedikt B. Kaufer

**Affiliations:** Institut für Virologie, Zentrum für Infektionsmedizin, Freie Universität Berlin, Robert-von-Ostertag-Straße 7-13, 14163 Berlin, Germany; andele.conradie@fu-berlin.de (A.M.C.); yuyou@zedat.fu-berlin.de (Y.Y.)

**Keywords:** lymphoma, avian cancer, herpesvirus, Marek’s disease virus, vaccine, life cycle, cell tropism

## Abstract

Marek’s disease virus (MDV) infects chickens and causes one of the most frequent cancers in animals. Over 100 years of research on this oncogenic alphaherpesvirus has led to a profound understanding of virus-induced tumor development. Live-attenuated vaccines against MDV were the first that prevented cancer and minimized the losses in the poultry industry. Even though the current gold standard vaccine efficiently protects against clinical disease, the virus continuously evolves towards higher virulence. Emerging field strains were able to overcome the protection provided by the previous two vaccine generations. Research over the last few years revealed important insights into the virus life cycle, cellular tropism, and tumor development that are summarized in this review. In addition, we discuss recent data on the MDV transcriptome, the constant evolution of this highly oncogenic virus towards higher virulence, and future perspectives in MDV research.

## 1. Introduction

Marek’s disease virus (MDV) mainly infects chickens and causes one of the most prevalent cancers in the animal kingdom [1,2]. The virus is strictly cell-associated and belongs to the genus Mardivirus in the subfamily Alphaherpesvirinae of the order Herpesvirales [3]. The Hungarian veterinarian József Marek first identified the disease in 1907 and described it as fowl paralysis, a generalized polyneuritis in chickens. His studies provided the basis for over 100 years of MDV research, which turned out to be an open-ended success story ‘from miasma to model’ [4,5].

MDV is among the diseases with the highest economic impact in modern poultry production worldwide [6], together with other viral diseases, such as Newcastle disease, infectious bronchitis and infectious bursitis [7]. Chickens are exposed to MDV around the globe and the virus is present in a large proportion of flocks [8]. The economic losses caused by MDV are due to lower feed conversion, weight loss, decreases in egg production, and condemnations of carcasses at slaughter [7,9]. In addition, the virus has an indirect economic impact by increasing the need for farming hygiene, vaccinations, and by inducing immunosuppression, which makes animals more susceptible to secondary infections [9,10]. Overall, MDV causes economic losses of about $1–2 billion in the poultry industry annually [11].

Widespread vaccination has drastically reduced the incidence of Marek’s disease [12]. However, MDV outbreaks are reported sporadically [13] and vaccine breaks do occur [14,15,16]. These events are most likely underestimated because Marek’s disease is not a notifiable disease in many countries [17], and the poultry industry is not interested in making disease outbreaks public. Moreover, vaccinated chickens are still susceptible to infection with MDV field strains and shed these into the environment [14]. These ‘imperfect’ vaccines allow the virus to evolve and acquire a higher virulence [18,19].

Aside from its importance in veterinary medicine, MDV infection of chickens is a versatile small animal model to study herpesvirus-induced disease, latency, and tumor formation in biomedical research. Overall, ‘avian tumor virology has laid the groundwork for much basic cancer research’ as acknowledged in ‘The oncologist’s debt to the chicken’ by R. A. Weiss [20]. Our review focuses on important recent discoveries in MDV infection, pathogenesis, and tumorigenesis, as well as the evolution towards higher virulence of this deadly pathogen.

## 2. Novel Insights into the MDV Life Cycle

**Figure 1:**
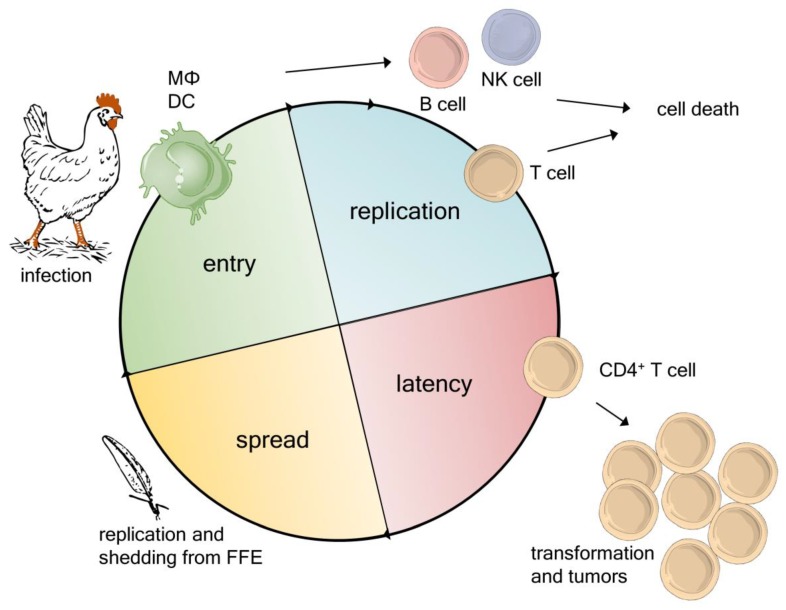
Marek’s disease virus (MDV) infection starts with the inhalation of infectious dust. Mononuclear phagocytes transfer the virus to lymphoid organs, such as the spleen, thymus, and bursa, where the virus lytically replicates in lymphocytes. MDV is able to establish latency in infected T cells. Latently and/or lytically infected T cells transport the virus to the skin and feather follicle epithelia (FFE), where cell free MDV is generated. In addition, MDV can transform latently infected T cells, resulting in deadly lymphomas.

The MDV life cycle is also known as the ‘Cornell model of MDV infection’ and was proposed by Calnek in 2001 [21]. The model describes a canonical order of events from initial infection to the generation of infectious virus in feather follicle epithelial cells [21]. The MDV life cycle can be broken down into four interlacing main phases: (i) entry, (ii) replication, (iii) latency, and (iv) spread (Figure 1). MDV infection starts with the inhalation of dust containing the infectious virus. Initial virus replication was previously detected in macrophages and B cells in the lung of infected animals [22]. Phagocytic cells, such as macrophages, are thought to transport the virus to regional lymphatic tissues, the bursa of Fabricius, and the spleen, where other immune cells become infected [14,21]. It has been shown that phagocytes-like macrophages and dendritic cells (DCs) support MDV replication and cell-to-cell spread in vitro [23] and infected chickens [24].

MDV secretes a viral CXC chemokine that was initially termed vIL-8, but recently changed to vCXCL13 based in its biological properties [25,26,27,28]. This chemokine recruits B cells and a subset of CD4+ T cells, and is crucial for the establishment of infection via the natural route [27]. The overall percentage of lytically infected cells in the primary lymphoid organs of infected chickens is generally rather low (below 1.2%), and only detectable between day 4–10 post infection [29,30,31]. Infection of chickens is accompanied by virus replication in several cell types, while B cells represent the majority of infected cells. Infected B cells can be readily detected in infected birds and appear to be the most susceptible cells for lytic replication [22,29,32].

However, we have recently demonstrated that B cells are completely dispensable for MDV pathogenesis and tumor formation [32]. In the absence of B cells, MDV can readily replicate in CD4+ and CD8+ T cells [32,33]. A recent study demonstrated that the virus can also replicate in other lymphocytes, such as natural killer (NK) cells, which are known to release interferon γ as an antiviral response [34]. Interestingly, interferon γ suppresses MDV replication and disease progression [35,36]. In addition, primary chicken endothelial cells were shown to be susceptible to infection [37]. These recent studies drastically increased our knowledge on the cell tropism of MDV and highlight that virus infection is not as sequential as previously assumed.

MDV not only replicates but also establishes latency in T cells [33,38,39]. Only a few latently infected cells are subsequently transformed, resulting in deadly lymphomas [40]. In latently infected and tumor cells, MDV integrates its viral genome into the telomeres of host chromosomes [41,42,43]. This integration ensures the maintenance of the virus genome with its oncogenes and is crucial for T cell transformation. Telomeric repeat arrays at the ends of the viral genome facilitate telomere integration, which likely occurs by a homologous recombination pathway [42]. The rapid formation of T cell lymphomas is the most characteristic feature of MDV infections. It has been shown that MDV-induced tumors consist mostly of T cells (> 60%), which represent, to a large extent, transformed and clonally expanded CD4+ T cells [40,44].

MDV can reactivate from the latently infected and tumor cells, allowing continuous virus shedding and spread to naïve individuals in the population [42]. Shedding occurs from feather follicle epithelial cells that release infectious dander and dust into the environment. Virus DNA can already be detected after 5–7 days post infection [18,45,46], while virus transmission starts between 12–14 days. Infectious virus is thought to be either encased in keratin or released by exocytosis [47] and remains infectious for 16–28 weeks [48,49], ensuring horizontal spread to naïve individuals (Figure 1).

## 3. Virulence Factors in the MDV Genome

The MDV double-stranded DNA genome is approximately 180 kb long and consists of short and long unique regions (U_S_ and U_L_), flanked by terminal repeats (TR_L_ and TR_S_), and internal repeats (IR_L_ and IR_S_) [50]. MDV encodes more than 100 genes that are involved in various processes of the viral lifecycle [51]. Among these are several virulence factors that act alone or in concert with each other to drive pathogenesis and tumor formation. These factors include the putative oncoprotein Meq, the viral chemokine vIL-8/vCXCL13, RLORF4, RLORF5a, pp14, and pp38, as reviewed recently [5,52].

Previous research has largely focused on the role of those viral protein-coding genes. However, MDV also encodes a rich repertoire of non-coding RNAs (ncRNAs) including micro RNAs (miRNAs) and a viral telomerase RNA (vTR) [53,54,55,56,57,58,59,60]. vTR shares 88% sequence identity and the conserved stem-loop structure with its cellular homolog in the chicken (cTR) [54]. Moreover, vTR is crucial for efficient virus-induced tumor formation [54]. Tumorigenesis of a virus lacking vTR could be restored by the insertion of chicken TR into the virus genome. These data highlight that overexpression of a cellular TR can drive tumor formation and that the virus likely acquired the gene from its host [61]. vTR can also be partially/completely complemented by two ncRNAs from the Epstein–Barr virus (EBV) that share cellular interaction partners that are highly conserved between chickens and humans [62]. Future studies should elucidate the transformation mechanism of vTR and its cellular homolog in this natural model for virus-induced tumor formation.

MDV also encodes miRNAs that play important roles during latency and tumorigenesis [59]. So far, 14 miRNA precursors were identified that produce 26 mature miRNAs. These miRNAs are located in three clusters within the MDV repeat regions and are known as the Meq-, Mid- and LAT-cluster [59,63]. They are involved in the regulation of viral [64,65] and cellular target genes [66]. Suppression of immediate early genes, for example, contributes to the establishment and maintenance of latency [67]. MDV-miR-M4 has an identical seed region as the human miRNA miR-155, and is one of the most highly expressed MDV-miRNAs [68,69]. Similar to human miR-155 [70], MDV-miR-M4 plays an important role in tumor formation, but it is not essential for the maintenance of the transformed phenotype and continuous proliferation of tumor cells [64,65,71].

Recent studies identified additional coding and non-coding sequences that are potentially important for infection, replication, pathogenesis, and spread [51,72]. For instance, they detected a plethora of novel splice variants, including variants of known genes, such as UL15 (MDV027), UL49 (MDV062), pp38 (MDV073), and pp24 (MDV008) [51]. Furthermore, RNA sequencing revealed novel poly(A) cleavage sites, polyadenylation signals, and polycistronic transcripts [51]. However, their relevance for the virus life cycle and their exact functions remain elusive [51,72]. In addition, we only have basic knowledge of post-translational modifications of MDV proteins, epigenetic modifications, and gene regulation [73].

## 4. MDV Evolution and Increase in Virulence

**Figure 2:**
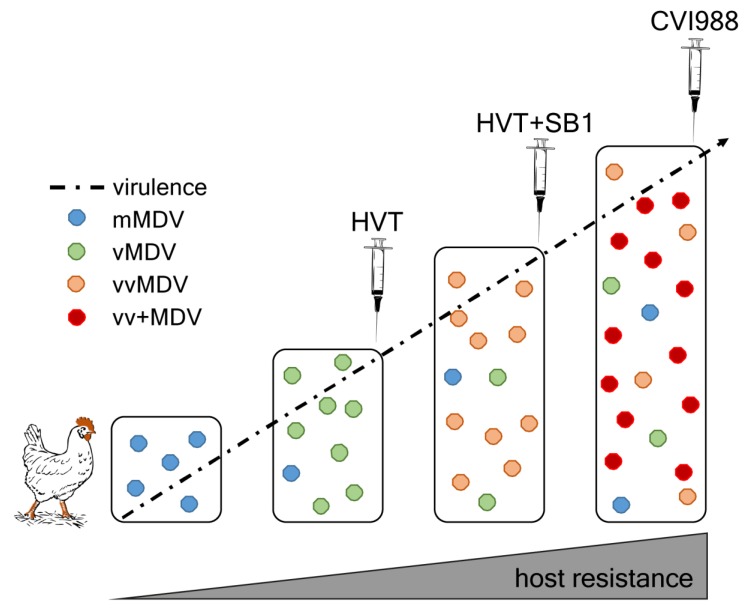
Increasing average virulence of MDV field strains and introduction of the different MDV vaccines over the past decades in the USA (HVT, herpesvirus of turkey; SB1, Gallid herpesvirus 3 strain; CVI988,non-oncogenic MDV strain). The HVT vaccine was launched in the 1970s, the bivalent HVT+SB1 in the late 1980s and the current CVI988 vaccine in the 1990s (USA)/1970s (Europe) [14]. Symbols represent the different MDV pathotypes: m (blue), v (green), vv (orange), and vv+ (red). At the same time, breeding programs focused on an increased resistance to MDV-induced tumorigenesis.

MDV remains a threat to the poultry industry due to its evolution towards higher virulence [74], with virulence defined as the ability of the virus to replicate, cause disease, affect host defense, and increase spread [75]. The MDV-induced disease has dramatically changed since its discovery, and virus strains are currently classified into four pathotypes based on their pathogenicity in experimental infections in vaccinated and unvaccinated chickens [76,77]. These range from mild (m), virulent (v), and very virulent (vv), to very virulent plus (vv+) (Figure 2, modified from [78]). The m strains only cause a rather mild neurological disease. The v strains, such as JM102, cause lymphomas with up to 40% mortality [79]. The vv strains, such as RB-1B or MD5, can cause a highly contagious lymphoproliferative disorder of chickens with many different clinical manifestations and high mortalities in unvaccinated flocks. The vv+ strains (e.g., 686) frequently cause severe brain edema and acute death within a few days in unvaccinated animals, and tumor lesions, even in vaccinated chickens [79,80,81,82,83]. Phylogenomic analyses revealed that this remarkable increase in virulence developed independently in Europe and North America [84]. However, the mechanism facilitating the evolution of MDV strains towards higher virulence remains largely unknown.

One potential selective pressure are the live-attenuated vaccines introduced since the 1970s to control MDV [85,86]. All MDV vaccines only prevent clinical symptoms, but do not prevent virus replication and shedding in the vaccinated host. While reduced shedding from vaccinated birds has been reported [87,88], these ‘imperfect’ vaccines still allow virus spread and evolution in the field, and are associated with the emergence of field strains with increased virulence (Figure 2) [14]. Mathematical modeling of key epidemiological parameters suggested that vaccination decreases mortality, but at the same time, does not significantly reduce the shedding rates [89]. Consistently, it has been recently shown that vaccination prolongs the survival of infected chickens. This provides vv+ strains enough time for efficient viral shedding and onward transmission [18], indicating that both shedding and transmission to naïve individuals represents a selective pressure. The efficiency of virus shedding is also influenced by chicken breeds, farm hygiene, and biosecurity [90].

Another potential driver of the increased virulence is the industrialization of poultry farming and intensive chicken husbandry [91,92]. A large number of animals (>20,000) in a confined space and the dramatically shortened average lifespan of chickens with industrialization allow efficient virus spread between individuals [93]. These intensive farming techniques, in combination with vaccination programs since the 1950s and 1970s, are the most plausible factors for the observed increase in virulence of circulating field strains [14,92].

During the evolution of MDV, specific changes in the genome have been documented that could contribute to the increase in virulence and allow the virus to overcome vaccine protection [74,84,94].

Comparative bioinformatic studies have been performed to identify genes that could contribute to the increase in MDV virulence. Mutations in several open reading frames, including *meq* (MDV076), ICP4 (MDV084), and ICP27 (MDV068) directly correlate to the changes in virulence [84]. Additional candidate genes are UL6 (MDV018), UL15 (MDV027), UL36 (MDV049), UL37 (MDV050), UL41 (MDV054), and R-LORF8 [74]. These genomic alterations could be used as pathotype markers in the future [74]. The strongest association with the observed increased virulence is the polymorphisms identified in the major oncoprotein and transcription factor Meq. Despite the rather low evolutionary rate of double-stranded DNA viruses [95,96], it has been reported that the *meq* gene is evolving at a much faster rate than most genes in double-stranded DNA viruses [97].

Taken together, the association of vaccine breaks and the increase in virulence in MDV infections seems to be a characteristic of MDV and MDV vaccines, rather than a common phenomenon.

## 5. Future Perspectives and Conclusions

Despite many years of MDV research, various challenges remain to be addressed. Firstly, the leaky MDV vaccines should be improved to provide better protection and to reduce virus shedding. Current vaccines also have immunosuppressive properties that should be eliminated [10]. In turn, this could enhance immune responses against other pathogens, as MD-induced immunosuppression leads to an increased susceptibility to *E. coli* and other pathogens [98,99]. A better understanding of MDV virulence factors will also allow a more focused development of novel MDV vaccines. Mechanisms of vaccine resistance should be addressed as this poses a constant risk to the poultry industry [18,19]. Moreover, future research should investigate why the ongoing evolution of virulence leading to failure of the CVI988 vaccine has not emerged as a significant industry problem yet. In addition, there is more to learn about the current ‘gold standard’ CVI vaccine that potentially integrates into the host chromosomes due to the presence of telomeric repeats (TMRs) in its genome [100]. In line with that, it has been recently suggested that MDV vaccines can also integrate into the host genome [101]. CVI stocks also encode at least two isoforms of the multifunctional Meq protein that fundamentally differ in their oncogenic potential [102]. Moreover, Meq has been reported to modulate intrinsic immune responses, including p53-mediated apoptosis [103] and the cGAS-STING pathway [104], but more work is needed to understand the molecular mechanisms of this key virulence factor.

Secondly, MDV infection in chickens will continue to be used as an animal model for herpesvirus research and virus-induced tumor formation. MDV can serve as a model to assess herpesviruses evolution [96] and to test pan-herpesvirus antiviral drugs [105]. Finally, researchers should further investigate the genetic resistance of the host to MDV infections [106,107,108,109]. In addition to conventional breeding programs, novel technology, including CRISPR/Cas9 genomic editing, could be used to generate more resistant chickens [110].

In summary, we highlighted the progress in MDV research from previous years, which provided intriguing insights into the lifecycle and cellular tropism of this highly oncogenic pathogen. Furthermore, recent studies shed light on an increasing coding capacity of the MDV genome and polymorphisms associated with increased virulence. Finally, we gained a better understanding of vaccine resistance, virus shedding, and an increase in virulence of this highly oncogenic herpesvirus.
